# Development of a Highly Efficient Multiplex Genome Editing System in Outcrossing Tetraploid Alfalfa (*Medicago sativa*)

**DOI:** 10.3389/fpls.2020.01063

**Published:** 2020-07-17

**Authors:** Tezera W. Wolabu, Lili Cong, Jong-Jin Park, Qinyan Bao, Miao Chen, Juan Sun, Bin Xu, Yaxin Ge, Maofeng Chai, Zhipeng Liu, Zeng-Yu Wang

**Affiliations:** ^1^Noble Research Institute, Ardmore, OK, United States; ^2^College of Grassland Science, Qingdao Agricultural University, Qingdao, China; ^3^College of Pastoral Agriculture Science and Technology, Lanzhou University, Lanzhou, China

**Keywords:** alfalfa, genome editing, CRISPR/Cas9, multiplex, mutagenesis, outcrossing, polyploid

## Abstract

Alfalfa (*Medicago sativa*) is an outcrossing tetraploid legume species widely cultivated in the world. The clustered regularly interspaced short palindromic repeats (CRISPR)/CRISPR-associated protein 9 (CRISPR/Cas9) system has been successfully used for genome editing in many plant species. However, the use of CRISPR/Cas9 for gene knockout in alfalfa is still very challenging. Our initial single gRNA-CRISPR/Cas9 system had very low mutagenesis efficiency in alfalfa with no mutant phenotype. In order to develop an optimized genome editing system in alfalfa, we constructed multiplex gRNA-CRISPR/Cas9 vectors by a polycistronic tRNA-gRNA approach targeting the *Medicago sativa* stay-green (*MsSGR*) gene. The replacement of CaMV35S promoter by the *Arabidopsis* ubiquitin promoter (AtUBQ10) to drive Cas9 expression in the multiplex gRNA system led to a significant improvement in genome editing efficiency, whereas modification of the gRNA scaffold resulted in lower editing efficiency. The most effective multiplex system exhibited 75% genotypic mutagenesis efficiency, which is 30-fold more efficient than the single gRNA vector. Importantly, phenotypic change was easily observed in the mutants, and the phenotypic mutation efficiency reached 68%. This highly efficient multiplex gRNA-CRISPR/Cas9 genome editing system allowed the generation of homozygous mutants with a complete knockout of the four allelic copies in the T0 generation. This optimized system offers an effective way of testing gene functions and overcomes a major barrier in the utilization of genome editing for alfalfa improvement.

## Introduction

Alfalfa (*Medicago sativa*), known as “Queen of forages”, is a widely cultivated perennial legume species with vast economic importance in the United States and other parts of the world. It is an obligate outcrossing tetraploid species and most genes are presented in multiple copies in the genome (Zhou et al., 2011; Gao et al., 2018). The existence of gene duplication (polyploidy) creates difficulties in various mutagenesis processes, and the outcrossing nature makes it very complicated to generate homozygous mutants. As a result, biotechnological studies in tetraploid alfalfa have been limited because of a lack of complete gene knockout (homozygous) mutants (Zhou et al., 2011; Ma et al., 2016; Gao et al., 2018). Therefore, the development of an efficient gene knockout system is highly desirable in alfalfa.

Clustered regularly interspaced short palindromic repeats (CRISPR)/CRISPR-associated protein 9 (Cas9) has become the most preferred genome editing tool, enabling continuous success in genetic manipulation of many crops (Voytas and Gao, 2014; Ma et al., 2016; Wang et al., 2016; Meng et al., 2017; Barry et al., 2018; Zhang et al., 2018; Erpen-Dalla et al., 2019; Jiang et al., 2019; Johansen et al., 2019; Tofazzal, 2019; Oh et al., 2020; Niu et al., 2020; Zhang et al., 2020). To date, several polyploid crops have been successfully edited by the gRNA-CRISPR/Cas9 genome editing system, including wheat (*Triticum aestivum*), cotton (*Gossypium hirsutum*), rapeseed (*Brassica napus*), and potato (*Solanum tuberosum*) (Wang et al., 2014; Sun et al., 2015; Wang et al., 2016; Andersson et al., 2017; Cai et al., 2017; Zhang et al., 2017; Kanazashi et al., 2018; Kusano et al., 2018; Johansen et al., 2019). However, in spite of such substantial genome editing achievements, the use of the CRISPR/Cas9 system in alfalfa is still very challenging compared to the improvements made in other dicot and monocot plants. For instance, the first reported CRISPR/Cas9 genome editing efficiency in alfalfa targeting the *SPL9* gene was only 2.2% with no mutant phenotype, and the authors remarked on the need to further enhance the efficiency of CRISPR/Cas9 in alfalfa (Gao et al., 2018). Hence, a robust and efficient CRISPR/Cas9 system is highly needed through a systematic optimization of Cas9 and gRNA expression levels.

CRISPR/Cas9 mutagenesis efficiency can be affected by several factors, including features of gRNA sequence, promoter driving Cas9 expression, concentration of Cas9-sgRNA complex, gRNA target sequence, assay methods, and time frame of culture incubation (Dang et al., 2015; Mikami et al., 2015; Xie et al., 2015; Wang et al., 2016; Dong et al., 2017; Xu et al., 2017; Gao et al., 2018; Zhang et al., 2018). A multiplex gRNA-CRISPR/Cas9 system based on tandem arrays of tRNA-gRNA sequences assembled in a clustered manner provides one of the most successful mutagenesis strategies in *Arabidopsis*, rice and wheat genome editing (Xie et al., 2015; Wang et al., 2016; Zhang et al., 2018). It has been also reported that CRISPR/Cas9 knockout efficiency was improved by modifying the chimeric structure of guide RNA duplex length and sequence at the end of fourth thymine to abolish the pausing effect of continuous thymine sequences on RNA polymerase III (Dang et al., 2015; Dong et al., 2017; Xu et al., 2017);.

In order to develop an optimized genome editing system in alfalfa, here we studied the effects of (1) multiplex gRNA verses single gRNA, (2) different promoters driving Cas9 and (3) different gRNA scaffold. The alfalfa stay-green gene (*MsSGR*) was used to analyze knockout efficiency phenotype, as plants with the *sgr* mutation show a visible greenish appearance during dark or shade treatment due to senescence induction (Ren et al., 2007; Zhou et al., 2011; Wu et al., 2016; Barry et al., 2018). Although the single gRNA-CRISPR/Cas9 system has been successfully used in a number of other plant species, this system is not effective in alfalfa. Our optimized multiplex gRNA-CRISPR/Cas9 system allows super-efficient genome editing in alfalfa as the mutagenesis efficiency is 30-fold higher than the single gRNA system. The effectiveness of our optimized genome editing system in alfalfa could be applicable in other legume species with complex genomes.

## Materials and Methods

### Plant Growth Conditions

Alfalfa genotype R2336 was vegetatively propagated in the greenhouse and used for genetic transformation. The CRISPR/Cas9 generated alfalfa mutants, wild type (control 1) and empty vector control (control 2) were grown under greenhouse conditions of 22°C/19°C day/night temperature, 16/8 h day/night photoperiod, 150 µmol∙m^−2^∙s^−1^ light intensity and 70% to 80% relative humidity. All alfalfa plants used for this study were propagated by vegetative cuttings to avoid the outcrossing problem.

### Multiplex gRNA-CRISPR/Cas9 Vector Construction and Transformation

**Figure 1:**
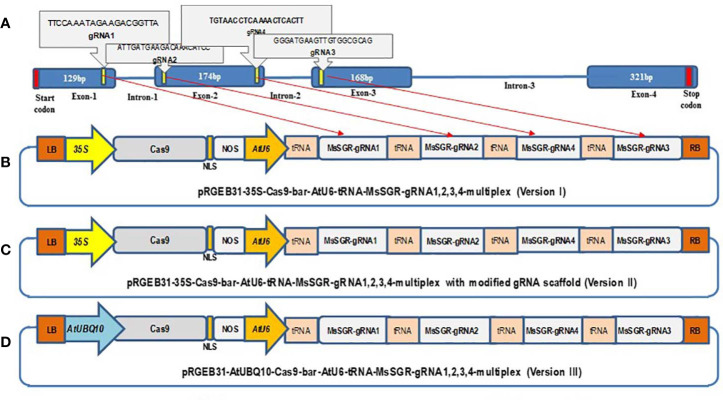
Schematic illustration of the stay-green (*MsSGR*) gene structure and the construction of different multiplex gRNA-CRISPR/Cas9 vectors for genome editing in alfalfa. **(A)** Alfalfa *MsSGR* gene structure and four designed gRNAs (1, 2, 3, 4) on exons 1, 2, and 3. **(B)** Map of a multiplex *MsSGR-*gRNA-CRISPR/Cas9 vector in which Cas9 is driven by the CaMV35S promoter (version I). **(C)** Map of a multiplex *MsSGR-*gRNA-CRISPR/Cas9 vector in which the gRNA scaffold is modified (version II). Red line at the 5′ of gRNAs of vector II indicates point mutation made at TTTTT to TTGTT to abolish the pausing effect of continuous T’s. **(D)** Map of a multiplex *MsSGR-*gRNA-CRISPR/Cas9 vector in which Cas9 is driven by the AtUBQ10 promoter (version III). All vector versions were constructed using polycistronic tRNA-CRISPR-gRNA (PTG) clustering system.

The *MsSGR* sequence was retrieved from the assembled alfalfa reference gene sequence DOBLAST (CADL) Genome Blast Server (https://www.alfalfatoolbox.org/doblast/?filterword=DOBLAST&function=function2) and http://blast.jcvi.org/Medicago-Blast/index.cgi based on the high sequence similarity between alfalfa and *M*. *truncatula* genomes. To avoid any potential genotypic SNPs that might affect genome editing through mismatch, the genomic DNA fragment was obtained by PCR amplification, cloned into pGEM-Teasy plasmids (TA-clone) (Promega, Madison, WI, USA) and subjected to sequence analysis. Four *MsSGR*-gRNAs were designed to target first, second, and third exon sites using the web-based tool CRISPR-P (http://cbi.hzau.edu.cn/cgi-bin/CRISPR) (Lei et al., 2014) (**Figure 1A**). The four 20-nts spacers were fused to gRNA scaffolds and clustered with tRNA in a tandem manner as described in the protocol given by Xie et al. (2015) using the Golden Gate assembly method (Engler et al., 2008). The pGTR plasmid, which contains a gRNA-tRNA fused fragment, was used as a template to synthesize polycistronic tRNA-gRNA (PTG) (Xie et al., 2015). To construct *MsSGR*-PTG, the gRNA scaffold fragment was amplified by PCR using a pair of specific primers (Bsal-*MsSGR*-gRNA1, 2, 3 and 4-F and *MsSGR*-gRNA1, 2, 3 and 4-R), whereas the tRNA fragment was amplified as a primer dimer of gRNA-F and tRNA-R and the fragments were fused as gRNA-tRNA by overlapping extension PCR using primers (**Supplementary Table 3**). The overlapping PCR products were separated and purified by the Spin Column PCR Product Purification Kit (Wizard SV Gel and PCR Clean-Up System) following manufacturer’s instructions (www.promega.com). Then the chain of multiplex tRNA-gRNA with four alfalfa spacers was inserted into three optimized vectors of pRGEB31 with AtU6-sgRNA, hSpCas9 backbone by digestion and ligation using Fok I (NEB) and BsaI enzyme step by step (Xie et al., 2015). We assembled three multiplex gRNA-CRISPR/Cas9 vectors: 1) AtU6-tRNA-gRNAs + 35S-Cas9, 2) AtU6-tRNA-gRNAs + 35S-Cas9 with modified gRNA scaffold sequence and 3) AtU6-tRNA-gRNAs + AtUBQ10-Cas9 (**Figures 1B–D**). CaMV35 (35S) was used to drive the expression of Cas9 in vector I; vector II was created by modifying the gRNA scaffold region with a point mutation according to the protocol given in Dang et al. (2015), whereas in vector version III, we replaced the 35S promoter by *Arabidopsis* ubiquitin promoter to drive Cas9 (**Figures 1B–D**). The subsequent multiplex gRNA-CRISPR/Cas9 binary vector modules were transferred into *Agrobacterium tumefaciens* and transgenic plants were obtained by *Agrobacterium*-mediated transformation method (Zhou et al., 2011). Briefly, fully developed trifoliate leaves were collected from 5- to 6-week-old alfalfa plants and sterilized; trifoliate leaf discs were infected with *A. tumefaciens* strain EHA 105 and co-cultivated for 24 to 36 h in the dark at 24 °C and then transferred onto selection media containing phosphinothricin. Resistant calli were produced and proliferated for five to six weeks. The resistant calli were then transferred onto a regeneration medium and cultured under light conditions of 150 µmol∙m^−2^∙s^−1^ at 24°C with 16/8 h photoperiod. PCR verification of the transgenic plants was conducted with genomic DNA extracted from leaf tissues using bar gene primers (**Supplementary Table 3**).

### Screening of *MsSGR* Mutants Generated by Multiplex gRNA-CRISPR/Cas9 in Alfalfa

To screen putative CRISPR mutants, genomic DNA was extracted from independent lines of alfalfa and the target region was amplified by PCR and then sequenced. Genomic DNA was extracted from young leaves of the regenerated lines of alfalfa using the CTAB method described by Rogers and Bendich (1988). Amplification of the target region was made by PCR using extracted genomic DNA as a template with specific primers designed from the border of the target site (**Supplementary Table 3**). The PCR products were treated with 1 µl of Antarctic Phosphatase and 0.25 µl Exonuclease I enzyme and incubated at 37°C for 1 h, PCR products were cleaned up by eliminating dNTPs residues. Then, 5 µl of PCR products were used for sequence analysis to verify CRISPR/Cas9-induced mutations in *MsSGR* transgenic lines. Furthermore, to identify and characterize the nature of mutation type induced by multiplex gRNA-CRISPR/Cas9 system at target sites (gRNA1, gRNA2, gRNA3 and gRNA4), the amplified fragments of mutated regions were sub-cloned into pGEM-T Easy plasmids. To precisely identify the Indels at the target site, 20 colonies were randomly selected and grown in LB medium for plasmid DNA isolation. The isolated single-colony DNA was subjected to Sanger sequencing for mutation events (Indels) analysis to determine homozygosity, heterozygosity and/or chimeric mutant. Reads were analyzed by aligning with the reference sequence using the SeqMan Pro 15.0.1 (DNASTAR software for life scientists) (https://www.dnastar.com/quote-request/).

### Alfalfa *sgr* Mutant Phenotype Screening by Dark/Shade-Induced Senescence

To decide whether the *MsSGR* mutants identified by genotyping screening could show a true stay-green phenotype, leaves collected from the mutants were exposed to dark-induced senescence treatment assay (Zhou et al., 2011). Typically the phenotype of a *sgr* mutant can easily be identified by its chlorophyll retention capacity using detached-leaf dark treatment or whole-plant shade treatment (Ren et al., 2007; Zhou et al., 2011; Xu et al., 2018). Replications of four to five leaves of *MsSGR* mutants and controls were harvested from the middle part of each plant and incubated at room temperature in darkness. The phenotype of leaf appearance (senescence) was evaluated every five days and photos were taken. At the 10th day of the incubation period, overall phenotypic efficiency was determined. This phenotypic screening procedure helps to confirm the effectiveness of the multiplex gRNA system in detail. Based on phenotypic grouping, *MsSGR* mutants were categorized into three classes: strong, mild and weak phenotype after 10 days of detached-leaf incubation in darkness.

## Results

### Single gRNA-CRISPR/Cas9 Mutagenesis Efficiency in Alfalfa

**Figure 2:**
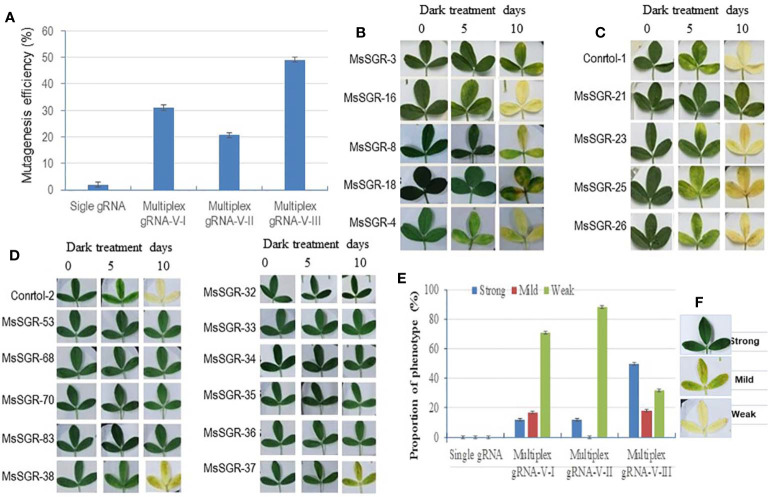
Mutagenesis efficiency and phenotype of alfalfa stay-green (*MsSGR*) mutants obtained by genome editing using different vectors. **(A)** Mutagenesis efficiency of single and multiplex gRNA-CRISPR/Cas9 vectors (versions I, II, and III) in *MsSGR* gene editing. **(B)** Phenotype of detached leaves of MsSGR mutants obtained by using multiplex vector version I during dark treatment assay. **(C)** Phenotype of detached leaves of MsSGR mutants obtained by using multiplex vector version II during dark treatment assay. **(D)** Phenotype of detached leaves of MsSGR mutants obtained by using multiplex vector version III during dark treatment assay. **(E)** MsSGR mutants’ phenotypic classes (strong, mild and weak) within three vector versions (I, II, and III) based on stay-green retention capacity during dark treatment (DT) assay. Control-1: wild type; control-2: empty vector control. **(F)** Detached leaf phenotypic categories of different alfalfa mutant lines after 10 days of dark treatment. Control-1: wild type; control-2: empty vector control. The detached leaf dark treatment assay in B-D was carried out at the same time using control-1 and contol-2 as common controls. Error bars indicate SE

As single gRNA-CRISPR/Cas9 has been successfully used in genome editing of many plant species, we originally constructed binary vectors using a single gRNA-CRISPR/Cas9 system for alfalfa genome editing (**Supplementary Figures 1A–E**). Alfalfa *stay-green* (*MsSGR*), *phytoene desaturase* (*MsPDS*) and other genes were used for target-specific mutagenesis. A total of 1,531 independent transgenic alfalfa lines were generated and examined for mutations (**Supplementary Table 1**). Unexpectedly, out of such a large number of transformants, no mutant phenotype was observed because there was no complete knockout of the four copies of the target genes. The overall genotypic mutagenesis efficiency was 2.5% with only one or two copies of the target gene mutated (**Figure 2A**; **Supplementary Table 1**). The complexity of alfalfa genome might be a reason for such a low efficiency of genome editing involving single gRNA-CRISPR/Cas9 approach. Furthermore, the outcrossing nature of this species made it impossible to obtain complete knockout in the next generation by selfing. Therefore, we had to change the genome editing strategy in alfalfa by testing different multiplex gRNA-CRISPR/Cas9 vectors.

### Multiplex gRNA-CRISPR/Cas9 Mutagenesis System in Alfalfa

Multiplex gRNA-CRISPR/Cas9 vectors were constructed to mutagenize all four allelic copies simultaneously. Four *MsSGR*-gRNAs were designed to target the first, second, and third exons of *MsSGR* (**Figure 1A**). Three vectors were constructed and tested (**Figures 1B–D**). In vector I (**Figure 1B**), Cas9 was driven by the CaMV35S promoter; vector II (**Figure 1C**) was obtained by modifying the gRNA scaffold region according to Dang et al. (2015); in vector III (**Figure 1D**), the CaMV35S promoter (used in vector I&II) was replaced by the *Arabidopsis* ubiquitin promoter (AtUBQ10) to drive Cas9.

A total of 492 transformants were obtained with the new multiplex vectors targeting the *MsSGR* gene. Genotypic analysis was performed by sequencing PCR products obtained by amplifying the target sites. Transgenic lines with mutations at the target sites were considered as candidate mutants of *MsSGR* and the ratio of mutagenized lines versus total examined lines across the populations was used as genotypic efficiency (mutagenesis efficiency). Genotyping analysis showed that the mutagenesis efficiencies were 31% (65 of 210), 23% (29 of 128) and 49% (71 of 144) for vector versions I, II and III, respectively (**Figure 2A**; **Supplementary Table 2**). Notably, the mutagenesis efficiency of vector III was higher than that of versions I and II (**Figure 2A**). Furthermore, the mutagenesis frequencies at four target sites differ across tested vectors (**Supplementary Figure 2**). For instance, vector version I showed 3.2%, 2.2%, 15.5% and 9.8% mutagenesis efficiencies for *MsSGR*-gRNA1, -gRNA2, -gRNA4 and -gRNA3, respectively (**Supplementary Figure 2**). Similarly, vector version II, the least efficient vector, showed 2.3%, 1.5%, 9.6% and 7.2% mutagenesis efficiencies for *MsSGR*-gRNA1, -gRNA2, -gRNA4 and -gRNA3, respectively (**Supplementary Figure 2**). On the other hand, the most effective vector, version III, showed 35%, 16%, 22% and 12% for *MsSGR*-gRNA1, -gRNA2, -gRNA4 and -gRNA3, respectively (**Supplementary Figure 2**).

The multiplex system was also tested for other genes, the efficiency of vector III was consistently higher than vectors I and II for every gene tested (**Supplementary Table 2**). The genotypic efficiency of vector III was in the range of 44% to 75% (**Supplementary Table 2**), which is 18 to 30 folds higher than the overall efficiency of the single gRNA-CRISPR/Cas9 system.

### Mutation Events Occurred in Genome Editing of *MsSGR* by the Multiplex gRNA-CRISPR/Cas9 System

**Figure 3:**
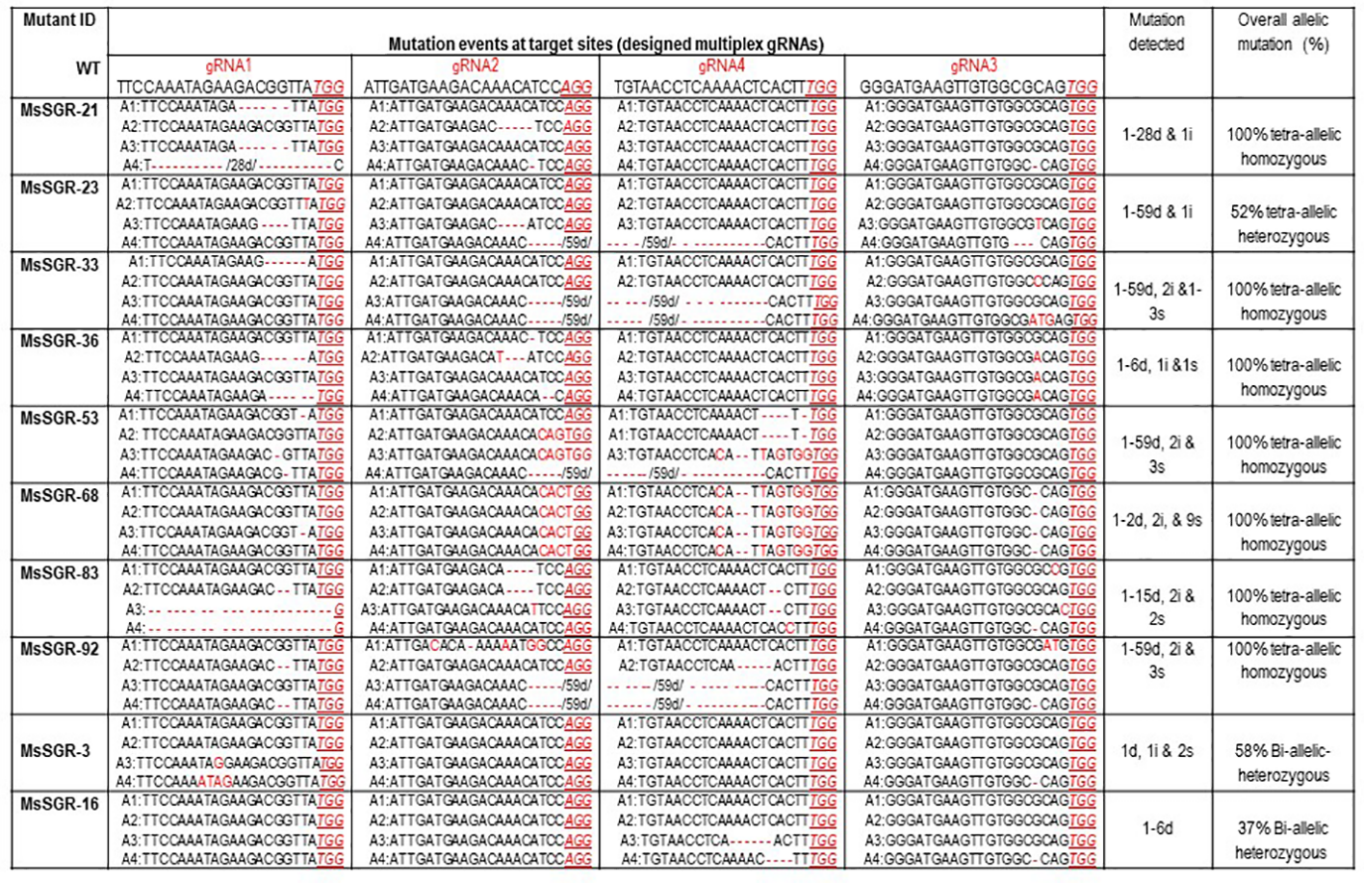
Molecular analysis of nucleotide deletion/insertion in MsSGR mutants generated by multiplex gRNA-CRISPR/Cas9 vectors. Mutation events detected at target sites (gRNA1, gRNA2, gRNA3, and gRNA4). Wild type (WT) sequence given for each target site (designed gRNA), deletions are indicated by red dashed lines with minus sign, insertions, or substitutions are indicated by red letters with plus sign and “s,” respectively, PAM indicated by red underlined italic letters. The four allelic copies of the MsSGR gene are designated as allele-1 (A1), allele-2 (A2), allele-3 (A3), and allele-4 (A4). Overall allelic copies mutated in percentage determine the homozygosity/heterozygosity of the mutant.

**Figure 4:**
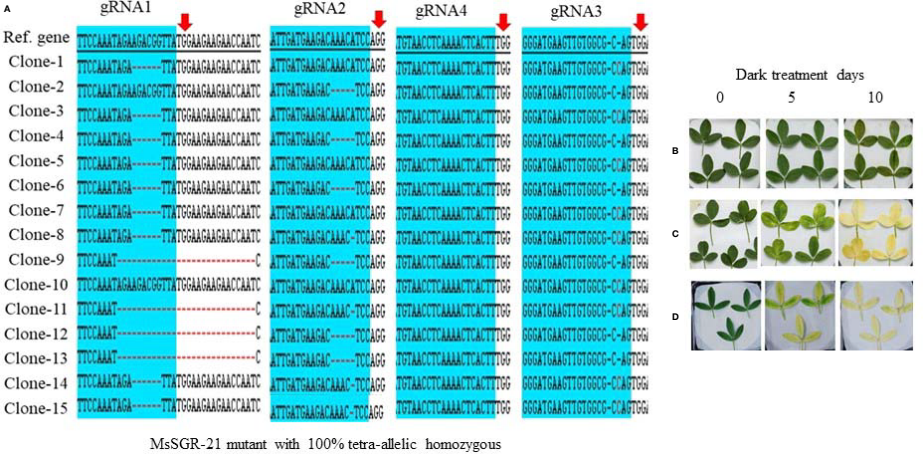
Genotypic and phenotypic analyses of *MsSGR* mutation by TA-clone sequencing and dark treatment. **(A)** Sequence analysis of different TA clones from MsSGR mutant #21 (MsSGR-21) at four target sites (gRNA1, 2, 3, and 4). Red arrow indicates the PAM of each gRNA, red dash indicates nucleotide deletion, red letter indicates insertion/substitution, colone-1, 2, … indicate corresponding plasmid sequences which represent allelic copies of the *MsSGR* gene in alfalfa. **(B)** Phenotype of detached leaves of MsSGR-21 mutant incubated in dark for 0, 5, and 10 days. Due to tetra-allelic homozygous mutation, all leaves stayed greenish. **(C, D)** Phenotype of detached leaves of wild type (WT) and empty vector control incubated in dark for 0, 5, and 10 days, all leaves became yellowish after ten days of dark incubation.

To identify and characterize mutation events induced in *MsSGR* by the multiplex gRNA-CRISPR/Cas9 system at the target sites (gRNA1, gRNA2, gRNA3 and gRNA4), each fragment of the mutated region was amplified and sub-cloned into pGEM-Teasy plasmid vector to determine the mutation events by Sanger sequencing. Twenty single colonies were randomly selected for each independent line to characterize the mutation events at the targeted sites (**Figures 3** and **4**; **Supplementary Figures 3A** and **4A**). The assessment of mutation events (deletion, insertion and substitution) at target regions also helps to determine the homozygosity/heterozygosity or chimeric nature of the mutant in depth. Ten *MsSGR* mutants, namely *MsSGR*-3 and *MsSGR*-16 for vector version I, *MsSGR*-21 and *MsSGR*-23 for vector version II, and *MsSGR*-33, *MsSGR*-36, *MsSGR*-53, *MsSGR*-68, *MsSGR*-83, and *MsSGR*-92 for vector version III, were subjected to this analysis (**Figure 3**). Different levels of nucleotide deletions (1–59 nts), insertions and substitutions (1–4 nts) at all target sites across the tested lines in alfalfa were detected (**Figure 3**; **Supplementary Figure 5**). Interestingly, one-nucleotide deletions, insertions and substitutions were the most frequent mutation events, with 37%, 26% and 20% occurrence, respectively, representing a total of 83% of overall mutation events (**Supplementary Figure 5**). Nucleotides “T” and “C” were most frequently involved in the insertion and/or substitution events, followed by “A” and “G.” In addition, large fragment deletions with 59 nts were the third most frequent mutation events, with 16% frequency between gRNA2 and gRNA4 in MsSGR-23, -33, -53, and -92 mutants (**Supplementary Figure 5**). Two- to 28 nts deletions were also detected across designed gRNAs with about 8% to 16% occurrence (**Supplementary Figure 5**). Notably, the occurrence of four induced allelic mutation copies was used to identify the homozygosity/heterozygosity of the line (**Figures 3** and **4A**; **Supplementary Figures 3A** and **4A**). For instance, the homozygosity of *MsSGR*-21 was the combination mutation events of occurred at gRNA1, gRNA2 and gRNA3 (**Figure 4**). This result revealed that sequences of all the clones have at least one or more mutation event at the target site(s), which made the mutant 100% tetra-allelic homozygous (**Figures 3** and **4A**). Furthermore, the results suggested the capability and effectiveness of at least two sgRNAs in multiplex CRISPR/Cas9 modules to knockout all allelic copies in tetraploid alfalfa genome.

### Phenotype Analysis of Alfalfa *sgr* Mutants

To determine whether the mutants display true stable stay-green phenotype, the mutants were subjected to dark-induced senescence assay. Three classes of phenotype (strong, mild and weak) were observed after detached leaves were incubated in darkness for 10 days (**Figures 2B–E** and **4B–D**; **Supplementary Figures 3B–D** and **4A–D**). Strong phenotype refers to the mutants with consistently retained greenish appearance throughout the incubation period (**Figures 2E, F**). Mild phenotype refers to the mutants with stay-green up to the fifth day that gradually changed to a mixture of light green and yellow (mosaic) by the 10th day of the incubation period (**Figures 2E, F**). Weak phenotype refers to those mutants that had light green color at the fifth day of dark incubation but became completely yellowish by the 10th day of the incubation period (**Figures 2E, F**). Interestingly, Vector III had the highest phenotypic efficiency, with 50%, 18% and 32% for strong, mild and weak phenotypes, respectively (**Figures 2E, F**; **Supplementary Table 2**). Vector I and II had a high proportion of weak phenotype (70% to 88%), with relatively low mild and strong phenotypic efficiency of 12% to 17% (**Figures 2E, F**). As illustrated in **Figures 2B–D**, *MsSGR*-18 and *MsSGR*-21 were the strong phenotype mutants generated by vector versions I and II, respectively (**Figures 2B, C**), whereas vector version III produced several strong mutants including *MsSGR*-53, *MsSGR*-68, *MsSGR*-70, *MsSGR*-83, *MsSGR*-32, *MsSGR*-33, *MsSGR*-34, *MsSGR*-35, and *MsSGR*-36 (**Figure 2D**).

Phenotype analysis was also carried out for other genes, vector III was consistently the most efficient one, with phenotypic efficiency in the range of 37% to 68% (**Supplementary Table 2**).

**Figure 5:**
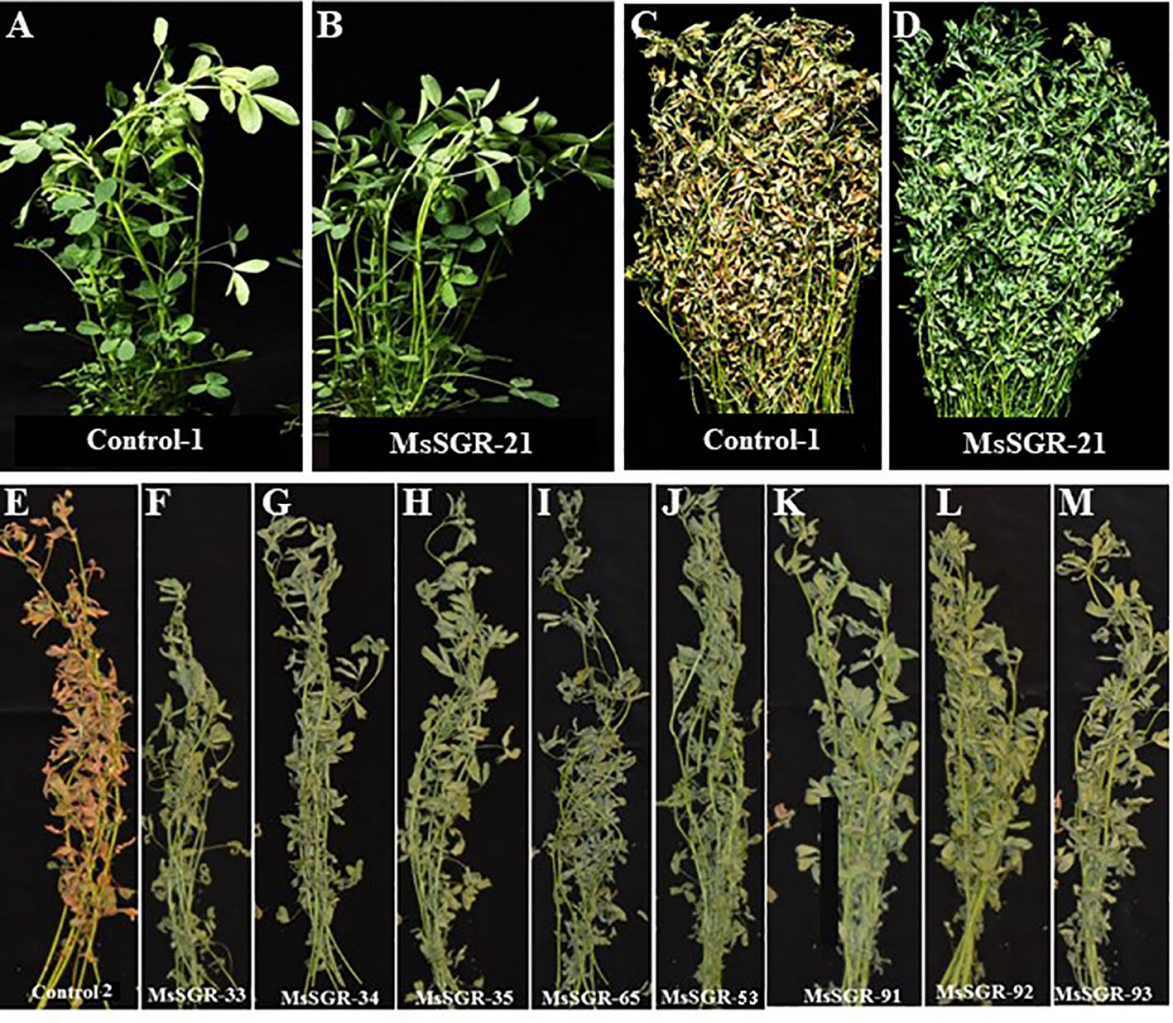
Illustration of phenotypic appearance of alfalfa *MsSGR* mutant harvested at first flower bud initiation stage and dried under the shade-induced condition for four weeks. **(A)** Phenotype of alfalfa wild type (WT) at vegetative stage (control), **(B)** Phenotype of MsSGR-21 mutant at vegetative stage, **(C)** Phenotypic appearance of alfalfa WT dried under shade for 4 weeks, **(D)** Phenotypic appearance of MsSGR-21 mutant dried under shade condition for four weeks shade, **(E)** Phenotypic appearance WT (control) (individual plant) dried under shade for four weeks, **(F–M)** Phenotypic appearance of eight independent lines of *MsSGR* mutant generated by multiplex gRNA-CRISPR/Cas9 vector version III after 4 weeks shade treatment. All *MsSGR* mutants showed strong phenotype due to their tetra-allelic homozygosity.

Similar to detached leaves dark treatment assay, when the whole plants of the strong mutants were kept under dry shade conditions (**Figure 5**), they consistently maintained the greenness after four weeks of shade treatment (**Figures 5D**, **F–M**). In contrast, the whole plants of wild type and empty vector became yellowish (bleached) after four weeks of shade treatment (**Figures 5C, E**). Taken together, phenotyping and genotyping analyses consistently revealed that the multiplex system is very effective in generating complete knockout mutants and version III is the most efficient vector for alfalfa genome editing.

### Alfalfa *sgr* Mutants Induced by Multiplex gRNA Showed Stronger Stay-Green Phenotype Than RNAi Lines

We further compared the effectiveness of *MsSGR* mutants generated by the multiplex gRNA-CRISPR/Cas9 system with previously developed *MsSGR*-RNAi transgenic lines (Zhou et al., 2011) using detached-leaf dark treatment assay. The multiplex gRNA-CRISPR/Cas9-generated mutants (*MsSGR*-21 and *MsSGR*-53) had excellent chlorophyll retention with a consistently deep green appearance throughout the dark incubation period, whereas the *MsSGR*-RNAi lines showed less strong phenotype with light greenish phenotype after 10 days of dark treatment (**Supplementary Figure 6**).

## Discussion

CRISPR/Cas9 has become a cutting-edge technology to provide an opportunity for more effective crop improvement than other commonly used methods (Cong et al., 2013; Mali et al., 2013; Xie et al., 2015; Wang et al., 2016; Park et al., 2017; Zhang et al., 2018; Erpen-Dalla et al., 2019; Jiang et al., 2019; Johansen et al., 2019; Tofazzal, 2019; Oh et al., 2020; Niu et al., 2020; Zhang et al., 2020). Regardless of substantial achievements in several crops, the efficiency of the CRISPR/Cas9 system in alfalfa is still challenging (Meng et al., 2017; Gao et al., 2018), which necessitate the development of a robust and effective CRISPR/Cas9 genome editing system. Initially, we spent a significant amount of time trying to establish an alfalfa mutagenesis system using single gRNA CRISPR/Cas9 because in our hands, such single gRNA system worked well in monocot species such as rice and switchgrass (Park et al., 2017). However, the overall mutagenesis efficiency in alfalfa was only 2.5%, which is close to a recently reported alfalfa genome editing efficiency of 2.2% (Gao et al., 2018). A more disappointing problem is that we did not find any mutant phenotype, indicating the no homozygous mutant was obtained. Again, this result is similar to the report by Gao et al. (2018) in which no phenotype was observed. The lack of mutant phenotype could be due to the presence of four allelic gene copies, resulting in incomplete knockout of the target gene. The outcrossing nature of alfalfa prevented us from obtaining homozygous mutants in the progeny by inbreeding.

By using a multiplex CRISPR/Cas9 system targeting different sites of the same gene simultaneously, we drastically increased genome editing efficiency, and more importantly, obtained complete knockout mutants with clear phenotypes. In dicot transformation and genome editing, CaMV35S promoter is the most commonly used promoter. Although the use of CaMV35S promoter to drive Cas9 (version I) allowed us to obtain a decent mutagenesis efficiency, significant increase in mutagenesis efficiency was obtained by replacing the CaMV35S promoter with the *Arabidopsis* ubiquitin promoter (version III). The tested multiplex CRISPR/Cas9 vector versions drastically improved alfalfa *stay-green* gene mutagenesis efficiency by 23% to 49% at the genotypic level. Remarkably, among the three multiplex vectors tested, version III is most effective, with genotypic efficiency reached 75%, which is 30 times more efficient than the single gRNA CRISPR/Cas9 system.

We also tested the effect of modification of the gRNA scaffold region with a point mutation (version II) because it was reported such an optimization led to large increase in genome editing efficiency (Dang et al., 2015; Zhang et al., 2018). However, in contrary to the enhanced mutation efficiency reported in previous studies (Dang et al., 2015; Zhang et al., 2018), the modification of the gRNA scaffold region led to decreased mutagenesis efficiency in alfalfa.

For phenotype assessment, we targeted the alfalfa *MsSGR* gene as a candidate to test the effectiveness, because mutants of the *stay-green* gene can be easily identified by dark-induced senescence. As reported in previous studies, *stay-green* gene knockdown or knockout mutants displayed greenness in leaves, stems, pods and seeds of the plants in *Arabidopsis thaliana*, *Medicago truncatula, Medicago sativa* (alfalfa) and *Lolium perenne* after induced senescence by dark treatment (Park et al., 2007; Ren et al., 2007; Zhou et al., 2011; Xu et al., 2018). After targeting four sites across the first, second, and third exons of the *MsSGR* gene, homozygous mutants were identified through rigorous genotypic and phenotypic analyses in the T0 generation. Various mutation events (deletions, insertions and substitutions) were found at the target sites, suggesting complete knockout of four allelic copies of the *MsSGR* gene, which led to obvious stay-green phenotype. Among the three multiplex vectors tested, version III is most effective in creating mutant phenotypes, the phenotypic mutation efficiency reached 68%.

Alfalfa is the fourth most widely grown crop in the United States behind only corn, wheat and soybean (Zhou et al., 2011). Most alfalfa produced in the US is used as hay, and visual appearance (greenish color) is an important trait for alfalfa hay (Zhou et al., 2011). A previous study showed that RNA-interference of *MsSGR* expression in alfalfa resulted in stay-green phenotype in dark-induced and natural leaf senescence and the effectiveness or degree of this stay-green phenotype was correlated with the RNAi suppression level of *MsSGR* (Zhou et al., 2011). In the current study, the homozygous *MsSGR* mutants generated by CRISPR/Cas9 showed more obvious greenish color than the RNAi lines, indicating complete knockout is more effective than RNAi downregulation in improving agronomic traits. This result demonstrated the success of an optimized multiplex gRNA-CRISPR/Cas9 system in alfalfa using a polycistronic gene construct cassette, which is simple and reliable as compared to previous biotechnology tools in alfalfa improvement.

In conclusion, we have developed a super-efficient multiplex gRNA-CRISPR/Cas9 genome editing system in alfalfa, which allowed the generation of homozygous mutants with a complete knockout of the four allelic copies in the T0 generation. Phenotypic change was easily observed in the mutants. This offers an effective way of testing gene functions and overcomes a major barrier in the utilization of genome editing for alfalfa improvement. The system may be used in genome editing of other dicot plants with complex genomes, particularly other legume species.

## Data Availability Statement

All datasets generated for this study are included in the article/**Supplementary Material**.

## Author contributions

Z-YW, TW, and ZL designed the research. TW, LC, J-JP, QB, MC, JS, BX, and YG performed the experiments and analyzed the data. TW and ZW wrote the paper. All authors contributed to the article and approved the submitted version.

## Conflict of Interest

The authors declare that the research was conducted in the absence of any commercial or financial relationships that could be construed as a potential conflict of interest.
