# Supplementary material for: Development of a Highly Efficient Multiplex Genome Editing System in Outcrossing Tetraploid Alfalfa (Medicago sativa)
Source: Front Plant Sci. 2020 Jul 17;11:1063. doi: 10.3389/fpls.2020.01063 (PMC7380066; doi:10.3389/fpls.2020.01063)
Supplement: Supplementary file 1 [file DataSheet_1.pdf]

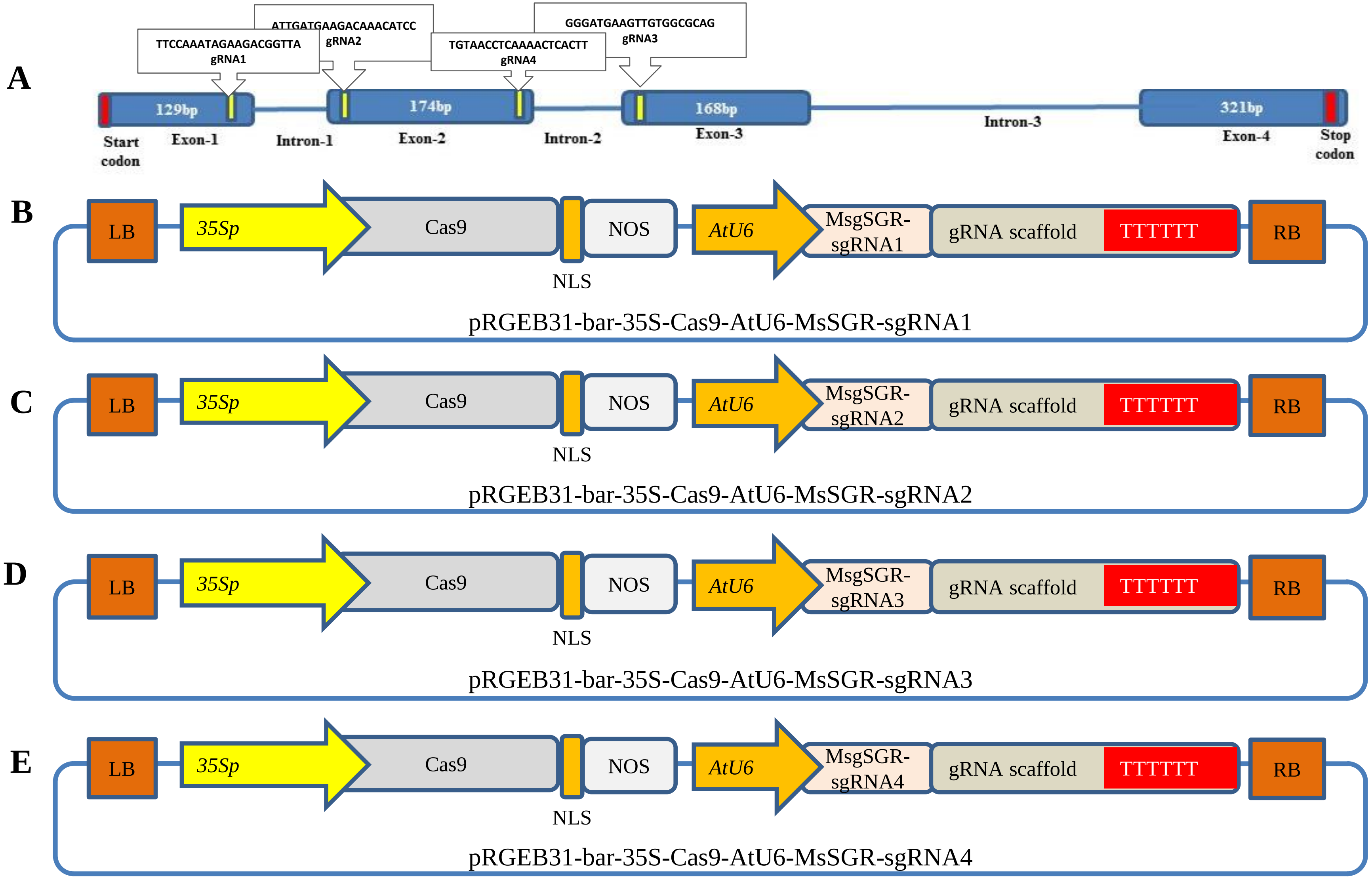

Supplementary Figure 1| Schematic illustration of *Medicago sativa* stay-green (*MsSGR)* gene structure and construction of single gRNA-CRISPR/Cas9 vectors for genome editing of *MsSGR* in alfalfa using each single gRNA one by one in vector construction. (**A**) *MsSGR* gene structure and four designed sgRNAs (1, 2, 3, 4) on exons 1, 2 and 3. (**B**) CRISPR/Cas9 vector using single gRNA1. (**C**) CRISPR/Cas9 vector using single gRNA2. (**D**) CRISPR/Cas9 vector using single gRNA3. (**E**) CRISPR/Cas9 vector using single gRNA4.

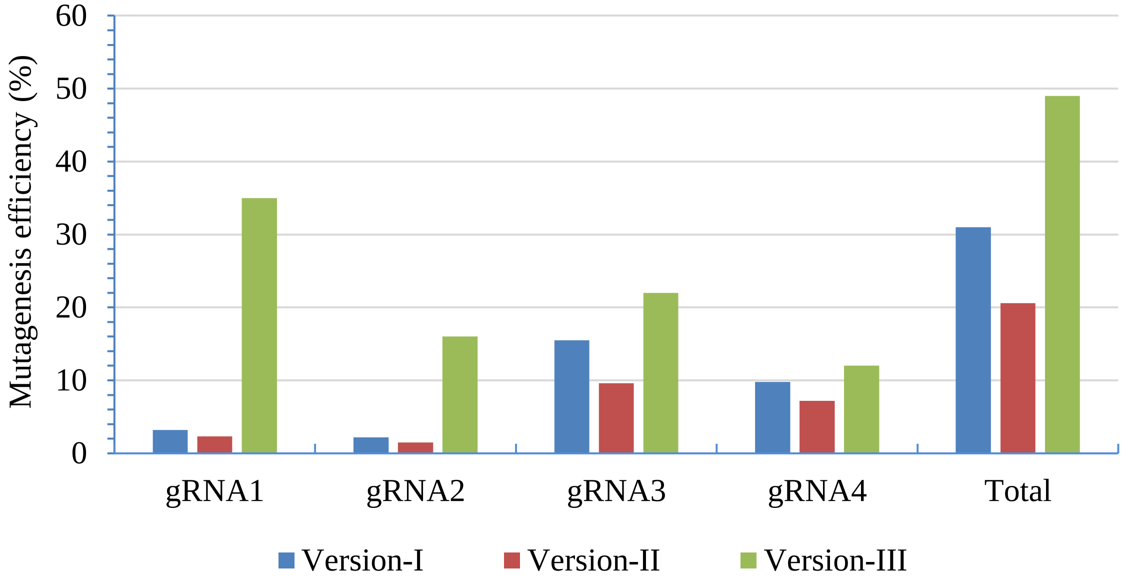

Supplementary Figure 2 | Mutagenesis efficiency of MsSGR gene editing using multiplex vector versions-I, II and III at each targeted site (gRNA1, 2, 3 and 4).

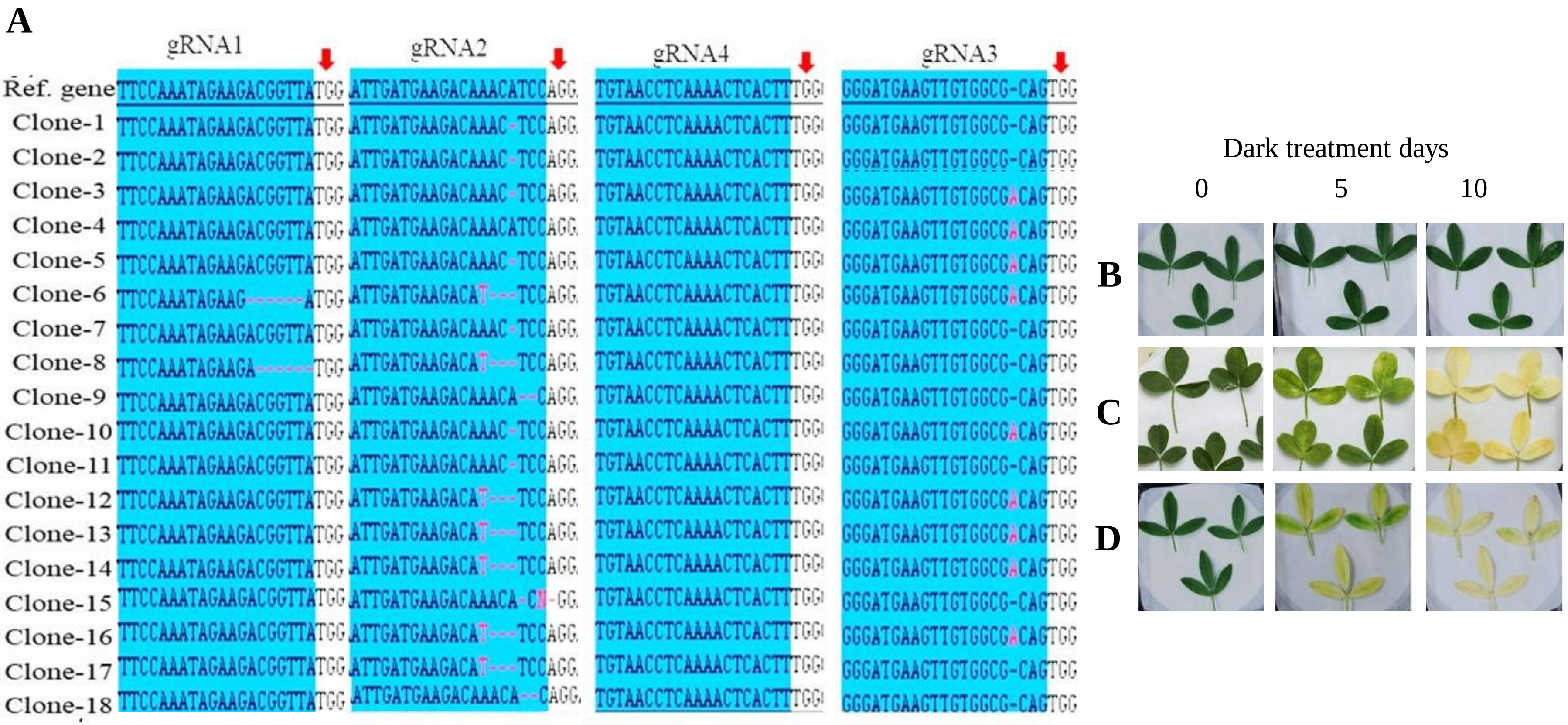

MsSGR-36 mutant with 100% tetra-allelic homozygous mutation

Supplementary Figure 3 | Genotypic and phenotypic analyses of *MsSGR* mutation by TA-clone sequencing and dark treatment. **(A)** Sequence analysis of different TA clones from MsSGR mutant #36 (MsSGR-36) at four target sites (gRNA1, 2, 3 & 4). Red arrow indicates the PAM of each gRNA, red dash indicates nucleotide deletion, red letter indicates insertion/substitution, colone-1, 2, … indicate corresponding plasmid sequences which represent allelic copies of the *MsSGR* gene in alfalfa. (**B**) Phenotype of detached leaves of MsSGR-36 mutant incubated in dark for 0, 5 and 10 days. Due to tetra-allelic homozygous mutation, all leaves stayed greenish. (**C&D**) Phenotype of detached leaves of wild type (WT) and empty vector control incubated in dark for 0, 5 and 10 days, all leaves became yellowish after ten days of dark incubation.

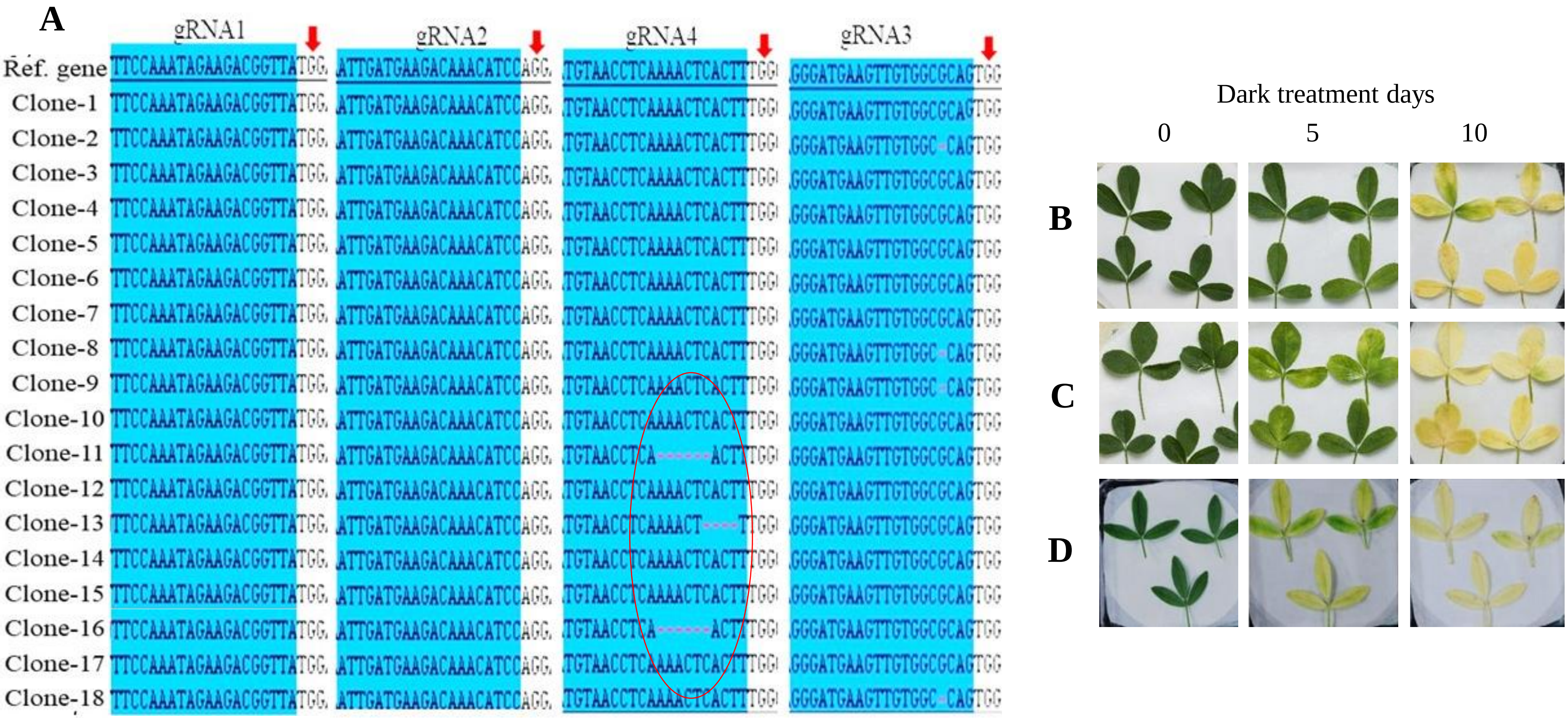

MsSGR-16 mutant with 37% tetra-allelic heterozygous/chimeric mutation

Supplementary Figure 4 |.Genotypic and phenotypic analyses of *MsSGR* mutation by TA-clone sequencing and dark treatment. (**A**) Sequence analysis of different TA clones from MsSGR mutant #16 (MsSGR-16) at four target sites (gRNA1, 2, 3 & 4). Red arrow indicates the PAM of each gRNA, red dash indicates nucleotide deletion, red letter indicates insertion/substitution, colone-1, 2, … indicate corresponding plasmid sequences which represent allelic copies of the *MsSGR* gene in alfalfa. (**B**) Phenotype of detached leaves of MsSGR-16 mutant incubated in dark for 0, 5 and 10 days. Due to heterozygous/chimeric mutation, degreening of leaves occurred at day 5 dark treatment, which is similar to the controls. (**C&D**) Phenotype of detached leaves of wild type (WT) and empty vector control incubated in dark for 0, 5 and 10 days, degreening of leaves started at day 5 dark treatment.

Mutation events	gRNA1	gRNA2	gRNA4	gRNA3	Total	Frequency (%)
1 nucleotides deletion	30	24	11	23	88	37.45
2 nucleotides deletions	4	11	10	0	25	10.64
3 nucleotides deletions	1	15	0	3	19	8.09
4 nucleotides deletions	0	0	5	0	5	2.13
5 nucleotides deletions	0	6	0	0	6	2.55
6 nucleotides deletions	32	0	6	0	38	16.17
15 nucleotides deletions	7	0	0	0	7	2.98
28 nucleotides deletions	14	0	0	0	14	5.96
59 nucleotides deletions	0	39	39	0	39	16.60
1 nucleotides insertion	3	12	2	45	62	26.38
1 nucleotides substitution	1	12	4	30	47	20.00
4 nucleotides substitutions	11	0	0	5	16	6.81

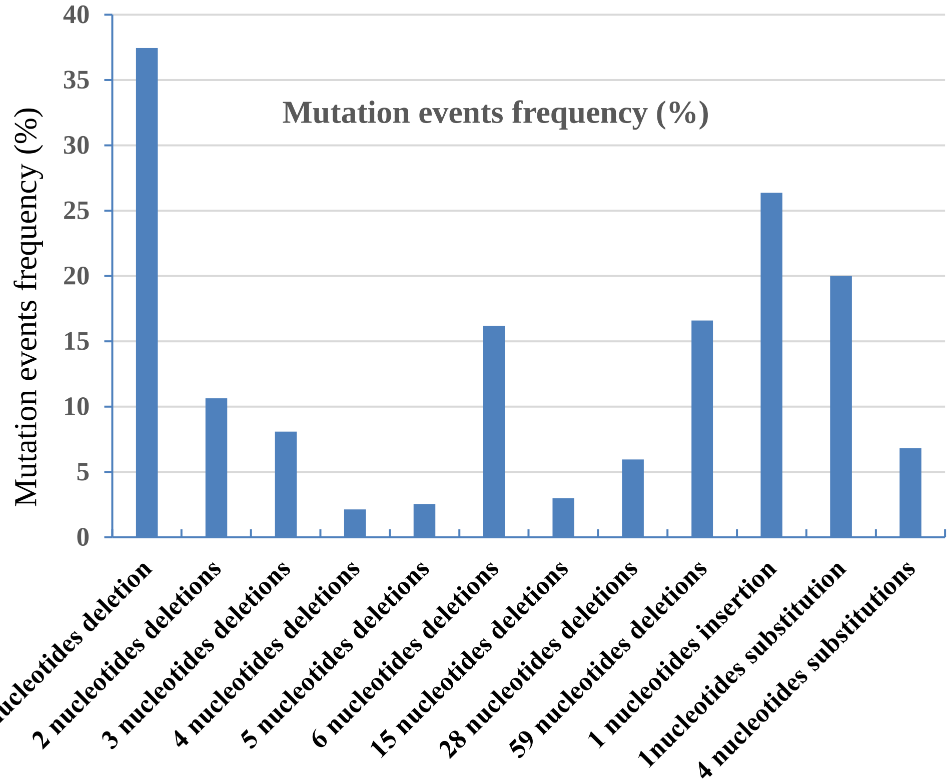

Supplementary Figure 5 | Status of mutation events occurrence in *MsSGR* at target sites. The percentage of mutation events frequency was calculated using the number of nucleotides deletions, insertions and substitutions that occurred at each target sites divided by the total number of TA clones of 10 mutants. Single-nucleotide sequence deletions and insertions were the most frequent mutation events in the multiplex gRNA-CRISPR/Cas9 system (n=235).

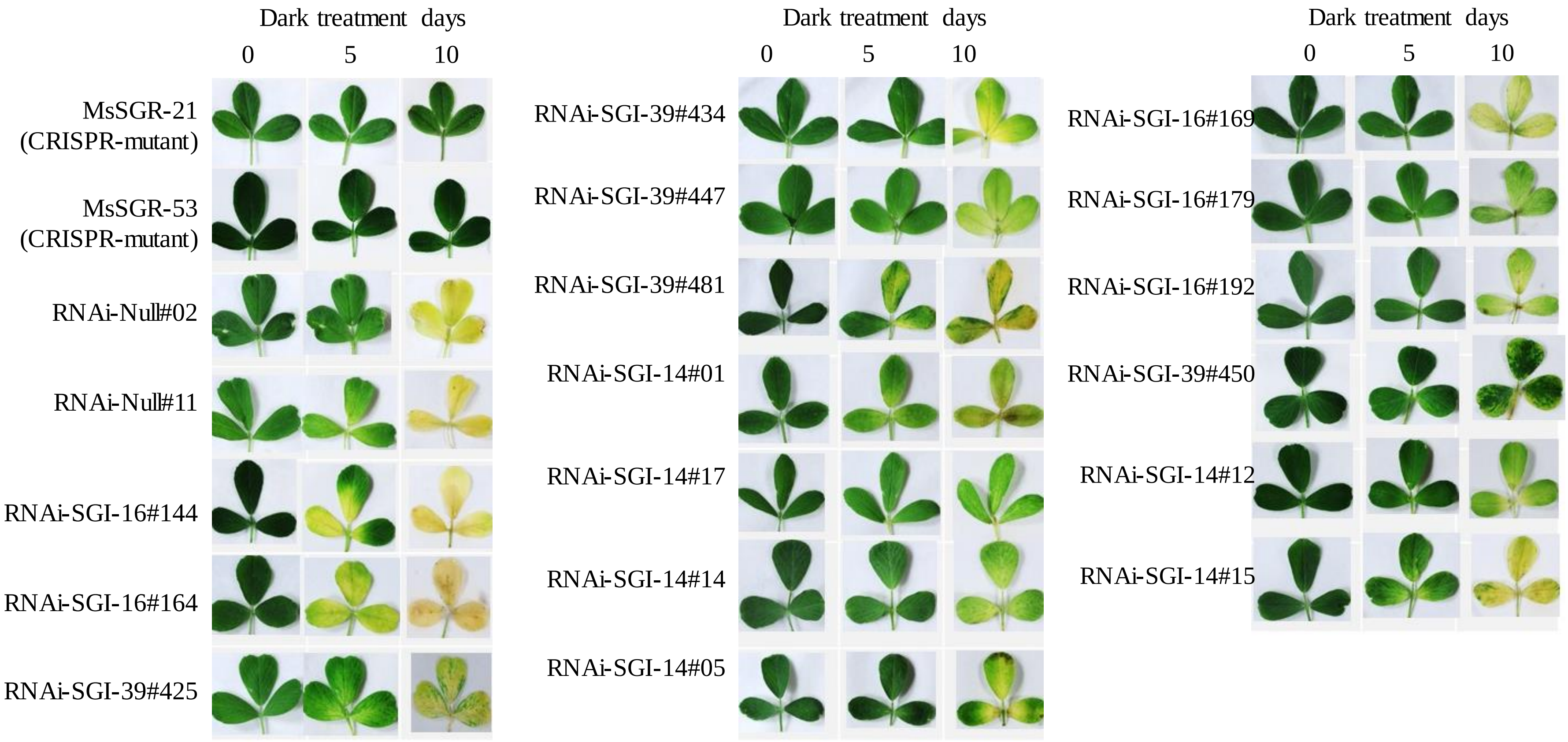

Supplementary Figure 6 | Phenotype of alfalfa stay-green (*MsSGR*) knockdown (RNAi) lines induced by dark treatment. Eleven MsSGR-RNAi transgenic lines showed mild phenotype with light green leaves after ten days of dark treatment, while four lines showed weak phenotype which is almost similar to the control. The two CRISPR/Cas9 generated mutants (MsSGR-21 and MsSGR-53) included in this dark treatment assay showed strong stay-green phenotype. Null#02 and Null#11 are empty vector controls.
