# Supplementary material for: Development of a Highly Efficient Multiplex Genome Editing System in Outcrossing Tetraploid Alfalfa (Medicago sativa)
Source: Front Plant Sci. 2020 Jul 17;11:1063. doi: 10.3389/fpls.2020.01063 (PMC7380066; doi:10.3389/fpls.2020.01063)
Supplement: Supplementary file 2 [file DataSheet_2.pdf]

Supplementary Table 1. Number of mutants generated and mutagenesis efficiency using single gRNA-CRISPR/Cas9 vector system for different alfalfa targeted genes

Name of gene	Designed gRNA	No. of examined lines	No. of mutants	Mutagenesis efficiency (%)	
				Genotypic efficiency	Phenotypic efficiency
MsSGR	gRNA1	131	11	8.4	None
	gRNA2	61	5	8.2	
	gRNA3	41	0	0	
	gRNA4	106	3	2.8	
MsPDS	gRNA1	380	0	0	None
	gRNA2	368	0	0	
MsFT	gRNA1	40	0	0	None
	gRNA2	40	0	0	
	gRNA3	40	3	0.7	
MsFD	gRNA1	20	0	0	None
	gRNA3	20	0	0	
MsC3H	gRNA1	72	4	5.5	None
	gRNA2	79	3	3.8	
	gRNA3	133	9	6.7	
Total	14	1531	38	2.5	

FT: Flowering locus T; FD: Flowering locus D; C3H: Coumarate 3-hydroxylase

Supplementary Table 2. Number of mutants generated and examined for mutagenesis efficiency using multiplex gRNA-CRISPR/Cas9 vector versions I, II and III targeting different alfalfa genes (N/A = not available)

Name of gene	No of examined lines				Genotypic efficiency (%)			Phenotypic efficiency (%)		
	Total	Version I	Version II	Version III	Version I	Version II	Version III	Version I	Version II	Version III
MsSGR	492	210	138	144	31	23	49	29	12	68
MsC3H	537	257	150	130	43	28	54	20	14	43
MsFTa	196	-	-	196	-	-	50	-	-	55
MsFD	48	-	-	48	-	-	44	-	-	37
MsPHO2	498	288	192	18	12	12	59	24	21	44
MsDASH1	343	130	158	55	74	56	75	-	-	
Total	2114	885	638	591	-	-	-	-	-	-

PHO2: PHOSPHATE2; DASH: DOF Acting in Seed embryogenesis and Hormone accumulation

Supplementary Table 3. List of targeting sequence and primers used in this study

Name	Sequence	Purpose	Name	Sequence	Purpose
**MsSGR-106-RTg3-F**	GAGTTGAAGAGGTTCACACA	For MsSGR mutant lines genotyping	L5AD5-F	CGGGTCTCAGGCAGGATGGGCAGTC<br>TGATTGaACAAAGCACCAGTGG	For Golden Gate vector assembly of multiplex tRNA-gRNA vector construction
**MsSGR-106-RTg3-R**	TATTCGCTAGATTAAGATATTTCA		L3AD5-R	TAGGTCTCCAAACGGATGAGCGAC<br>AGCAAACAAAAAAAAAGCACCGACTCG	
**MsSGR-107-RTg124-F**	CTATGCTCACTTCTAAGTTCAA		S5AD5-F	CGGGTCTCAGGCAGGATGGGCAGTCTGATTG	
**MsSGR-107-RTg124-R**	AAACTCACTTTGGCAATTTCT		S3AD5-R	TAGGTCTCCAAACGGATGAGCGACAGCAAAC	
**MsSGR-gRNA1-F**	TTCCAAATAGAAGACGGTTA	MsSGR site-1, 2, 3 & 4 single gRNA vector construction	Hyg-F	AAGGAATCGGTCAATACACTACATGG	Hyg selection marker
**MsSGR-ssgRNA1-R**	TAACCGTCTTCTATTTGGAA		Hyg-R	AAGACCAATGCGGAGCATATACG	
**MsSGR-sgRNA2-F**	ATTGATGAAGACAAACATCC		MsSGR-gRNA-1-F	TAGGTCTCCAGAAGACGGTTAGTTTTAGAGCTAGAA	For MsSGR multiplex tRNA-gRNA-CRISPR/Cas9 vector construction
**MsSGR-sgRNA2-R**	GGATGTTTGTCTTCATCAAT		MsSGR-gRNA-1-F	CGGGTCTCATTCTATTTGGAATGCACCAGCCGGG	
**MsSGR-sgRNA3-F**	GGGATGAAGTTGTGGCGCAG		MsSGR-gRNA-2-F	TAGGTCTCCAGACAAACATCCGTTTTAGAGCTAGAA	
**MsSGR-sgRNA3-R**	CTGCGCCACAACTTCATCCC		MsSGR-gRNA-2-F	CGGGTCTCAGTCTTCATCAATTGCACCAGCCGGG	
**MsSGR-sgRNA4-F**	TGTAACCTCAAAACTCACTT		MsSGR-gRNA-3-F	TAGGTCTCCGTTGTGGCGCAGGTTTTAGAGCTAGAA	
**MsSGR-sgRNA4-R**	AAGTGAGTTTTGAGGTTACA		MsSGR-gRNA-3-F	CGGGTCTCACAACTTCATCCCTGCACCAGCCGGG	
**MsPDS-sgRNA1-F**	GTATCATCAAGCTCAGGACG	MsPDS target site-1& 2 sgRNA vector	MsSGR-gRNA-4-F	TAGGTCTCCCAAAACTCACTTGTTTTAGAGCTAGAA	
**MsPDS-sgRNA1-R**	CGTCCTGAGCTTGATGATAC		MsSGR-gRNA-4-F	CGGGTCTCATTTGAGGTTACATGCACCAGCCGGG	
**MsPDS-sgRNA2-F**	GAGGCAAGAGATGTTCTAGG		Ms PHO2-gRNA-1-F	TAGGTCTCCCAGTGAGAGCAGGTTTTAGAGCTAGAA	For MsPHO2 multiplex tRNA-gRNA-CRISPR/Cas9 vector construction
**MsPDS-sgRNA2-R**	CCTAGAACATCTCTTGCCTC		Ms PHO2-gRNA-1-F	CGGGTCTCAGCTGCTCTCACTTGCACCAGCCGGG	
**MsU6-F**	AAGTGCAGAGTTTACAGTAACTAAAATCATGG	MsU6 promoter region	Ms PHO2-gRNA-2-F	TAGGTCTCCAACATTTGCTTAGTTTTAGAGCTAGAA	
**MsU6-R**	AAGCCCTGTTGTTCGCCTG		Ms PHO2-gRNA-2-F	CGGGTCTCAGTAAGCAAATGTTGCACCAGCCGGG	
**35S-F**	ACAGTCTCAGAAGACCAAAGGGC	35S promoter region	Ms PHO2-gRNA-3-F	TAGGTCTCCTTGTTTGATGACGTTTTAGAGCTAGAA	
**35S-R**	TGTTCTCTCCAAATGAAATGAAC		Ms PHO2-gRNA-3-F	CGGGTCTCAGTCATCAAACAATGCACCAGCCGGG	
**AtUBQ10-F**	CTGCAGGTCGACGAGTCAGTAATA	AtUBQ10 promoter region	MsDASH1-gRNA1-F	TAGGTCTCCCAGAAGTGTCCTGTTTTAGAGCTAGAA	For MsDASH1 multiplex tRNA-gRNA-CRISPR/Cas9 vector construction
**AtUBQ10-R**	TGTTAATCAGAAAAACTCAGATTAAT		MsDASH1-gRNA1-R	CGGGTCTCATCTGAGGTTGAGTGCACCAGCCGGG	
**AtU6-F**	GTGATTGTGAGACCGAGAG	AtU6 promoter region	MsDASH1-gRNA2-F	TAGGTCTCCGTACTGGACTCAGTTTTAGAGCTAGAA	
**AtU6-R**	CTGATAACTCTGATGTGGATAAG		MsDASH1-gRNA2-R	CGGGTCTCAGTACCTTCTGCATGCACCAGCCGGG	
**PPT-F (bar)**	GAAGTCCAGCTGCCAGAAAC	PPT selection marker	MsDASH1-gRNA3-F	TAGGTCTCCCCATGGCTGCGAGTTTTAGAGCTAGAA	
**PPT-R (bar)**	AGTCGACCGTGTACGTCTCC		MsDASH1-gRNA3-R	CGGGTCTCAATGGTCAACTGGTGCACCAGCCGGG	
**MsFTa-gRNA1-F**	TAGGTCTCCCCACTGGCTGTAGTTTTAGAGCTAGAA	For MsFTa multiplex tRNA-gRNA-CRISPR/Cas9 vector construction	MsDASH1-gRNA4-F	TAGGTCTCCTGTCGCCTTTTGGTTTTAGAGCTAGAA	
**MsFTa-gRNA1-R**	CGGGTCTCAGTGGATTCCTACTGCACCAGCCGGG		MsDASH1-gRNA4-R	CGGGTCTCAGACATGTAACCATGCACCAGCCGGG	
**MsFTa-gRNA2-F**	TAGGTCTCCCAGAGTGAGTGTGTTTTAGAGCTAGAA		MsC3H-gRNA1-F	TAGGTCTCCACTTCCCATCATGTTTTAGAGCTAGAA	For MsC3H multiplex tRNA-gRNA-CRISPR/Cas9 vector construction
**MsFTa-gRNA2-R**	CGGGTCTCATCTGGGTTGATTTGCACCAGCCGGG		MsC3H-gRNA1-R	CGGGTCTCAAAGTGGGTATGGTGCACCAGCCGGG	
**MsFTa-gRNA3-F**	TAGGTCTCCACCCCACTTTTAGTTTTAGAGCTAGAA		MsC3H-gRNA2-F	TAGGTCTCCCTCAAACATCTAGTTTTAGAGCTAGAA	
**MsFTa-gRNA3-R**	CGGGTCTCAGGGTTACTTGGGTGCACCAGCCGGG		MsC3H-gRNA2-R	CGGGTCTCATGAGAGTTTTGTTGCACCAGCCGGG	
**MsFTa-gRNA4-F**	TAGGTCTCCGACTGATATTCCGTTTTAGAGCTAGAA		MsC3H-gRNA3-F	TAGGTCTCCTTGATATTGGTGGTTTTAGAGCTAGAA	
**MsFTa-gRNA4-R**	CGGGTCTCAAGTCACCAACCTTGCACCAGCCGGG		MsC3H-gRNA3-R	CGGGTCTCATCAAAAACCTCATGCACCAGCCGGG	
**MsFD-gRNA1-F**	TAGGTCTCCATGGAAGAAGTTGTTTTAGAGCTAGAA	For MsFD multiplex tRNA-gRNA-CRISPR/Cas9 vector construction	MsC3H-gRNA4-F	TAGGTCTCCCATGGAAGAAGCGTTTTAGAGCTAGAA	
**MsFD-gRNA1-R**	CGGGTCTCACCATGGTTAATTTGCACCAGCCGGG		MsC3H-gRNA4-R	CGGGTCTCACATGATACCCCATGCACCAGCCGGG	
**MsFD-gRNA2-F**	TAGGTCTCCCACTTTTGGCGGGTTTTAGAGCTAGAA		MsDASH1-RT-F	AGAGATGGAGCAAGAACG	For MsDASH1 mutant genotyping
**MsFD-gRNA2-R**	CGGGTCTCAAGTGGAATTACGTGCACCAGCCGGG		MsDASH1-RT-R	ACACCAACATTCTGAGACTG	
**MsFD-gRNA3-F**	TAGGTCTCCACAACACAACATGTTTTAGAGCTAGAA		MsFD-RT-F	TCACAAATCAAACCCTCAATAG	For MsFD mutant genotyping
**MsFD-gRNA3-R**	CGGGTCTCATTGTTGTTGGTGTGCACCAGCCGGG		MsFD-RT-R	AACGTGCAGCAGATTCC	
**MsFD-gRNA4-F**	TAGGTCTCCTGATCAGAATGTGTTTTAGAGCTAGAA		MsFTa-RT-F	AGATTGGTACGCAACAGG	For MsFD mutant genotyping
**MsFD-gRNA4-R**	CGGGTCTCAATCATACGGTGATGCACCAGCCGGG		MsFTa-RT-R	AAAGTCACAAATGAAAGAATAGTT	
**MsPHO2-RT-F**	ATGGAAGCTCAGATGACTGATTCA	For MsPHO2 mutant genotyping	AtU6-region-RT-F	TTCAAAAGTCCCACATCGC	For vector versions plasmid colony genotyping
**MsPHO2-RT-R**	CAGAACAAACAGTTCCTTCATCATG		sgRNA-scaffold-RT-R	AAGGCGATTAAGTTGGGTAA	
**MsC3H-RT-F**	CTATTAGTGATTACCTCTTTGTGT	For MsC3H mutant genotyping	AtU6-region-RT-F2	GTGATTGTGAGACCGAGAG	
**MsC3H-RT-R**	AAATCTCACACAACTTTTTCATA		sgRNA-scaffold-RT-R2	CTGATAACTCTGATGTGGATAAG	
